# Prehypertension increases the risk of atherosclerosis in drug-naïve Japanese patients with type 2 diabetes mellitus

**DOI:** 10.1371/journal.pone.0201055

**Published:** 2018-07-20

**Authors:** Ippei Kanazawa, Toshitsugu Sugimoto

**Affiliations:** Department of Internal Medicine 1, Shimane University Faculty of Medicine, Izumo, Shimane, Japan; International University of Health and Welfare, School of Medicine, JAPAN

## Abstract

**Purpose:**

Hypertension is a risk factor of atherosclerotic diseases. However, the importance of prehypertension in Japanese patients with type 2 diabetes mellitus (T2DM) is controversial. The aim of this study was to examine association between prehypertension, hypertension and atherosclerosis in T2DM.

**Methods:**

We recruited 179 Japanese patients with T2DM, who never took any medication for diabetes, hypertension, dyslipidemia, or atherosclerosis. Intima-media thickness (IMT) of common carotid artery was evaluated by high-resolution B-mode ultrasonography.

**Results:**

Multiple regression analysis adjusted for age, duration of diabetes, body mass index, HbA1c, fasting C-peptide, triglyceride, HDL-cholesterol, LDL-cholesterol, and estimated glomerular filtration rate showed that systolic blood pressure (SBP), but not diastolic BP, was significantly and positively associated with maximum IMT (IMT-max), mean IMT, and plaque score (β = 0.28, p<0.001; β = 0.26, p = 0.047; and β = 0.25, p = 0.006, respectively). ROC analysis showed that the cut-off value of SBP to detect atherosclerosis (IMT-max 1.8mm, the mean of IMT-max of this subjects) was 133.5 (p = 0.008), while DBP was not useful to detect it (p = 0.433). Then, participants were categorized as normotension (SBP <119 mmHg), prehypertension (SBP 120–139 mmHg), and hypertension (>140 mmHg). Multiple logistic regression analysis adjusted for the variables described above plus gender and smoking showed that prehypertension and hypertension were significantly associated with the increased risk of atherosclerosis [prehypertension; odds ratio (OR) 3.45, 95% confidence interval (CI) 1.11–10.76, p = 0.033, and hypertension; OR 7.29, 95%CI 1.99–26.78, p = 0.003].

**Conclusion:**

These findings suggest that prehypertension categorized by SBP is an important risk factor of atherosclerosis independently of conventional risk factors in patients with T2DM.

## Introduction

Diabetes mellitus is known to increase atherosclerotic diseases. Previous studies have shown that the incidence of cardiovascular diseases (CVD) in patients with type 2 diabetes mellitus (T2DM) is two to three times higher than that in age-matched nondiabetics [1,2]. However, previous large-scale interventional studies have shown that intensive glycemic control alone could not completely decrease the risk of CVD events and mortality in patients with T2DM [3–5]. In contrast, an intensified, multifactorial intervention for hyperglycemia, hypertension, dyslipidemia, and microalbuminuria significantly reduced the risk of microvascular and CVD events [6] as well as mortality [7] compared to conventional treatment. Previously, UKPDS38, a randomized controlled trial comparing tight control of BP [less than systolic BP (SBP) 150 and diastolic BP (DBP) 85 mmHg] with less tight control (less than SBP 180 and DBP 105 mmHg) on macro- and microvascular diseases in patients with T2DM accompanied by hypertension showed that tight BP control significantly reduced the risk of stroke [hazard ratio (HR) 0.56, p = 0.013] but not myocardial infarction (p = 0.13) [8]. Because several studies have shown that prehypertension (SBP 120–139 or DBP 80–90 mmHg) increases the risk of CVD events in T2DM [9–11], further BP titration was believed to be beneficial for preventing CVD and stroke events in diabetic patients. However, ACCORD BP, a randomized trial performed in United States and Canada, showed that intensive therapy for BP, targeting less than SBP 120 mmHg, as compared to less than 140 mmHg, did not reduce the primary composite cardiovascular outcome, although stroke was significantly reduced (HR 0.59, p = 0.01) [12]. Therefore, there are still conflicting views on the therapeutic target of BP to prevent macrovascular diseases in T2DM.

Several studies suggest that there are ethnic differences in the association between BP and stroke between Asian and Caucasian. Eastwood et al. showed that the combination of hypertension and hyperglycemia was particularly detrimental for Asian patients, compared to European [13]. Li et al. recently reported a meta-analysis showing that the incidence of major cerebrovascular events was significantly greater in Asian than Western patients with T2DM, whereas the incidence of major coronary events and cardiovascular death was significantly lower in Asians [14]. Most recently, a large-scale interventional prospective study to examine the effects of an intensified, multifactorial intervention for blood glucose, BP, and serum lipids on cardiovascular outcomes and mortality, compared to conventional treatment, in Japanese patients with T2DM was reported [15]. In the study, intensive treatment did not significantly reduced the primary cardiovascular outcome (myocardial infarction, revascularization, stroke, and all-cause mortality) (p = 0.094); however, a post-hoc analysis of the composite outcome showed that cerebrovascular events were significantly less frequent in the intensive therapy group (targeting BP was less than SBP 120 and DBP 75 mmHg), compared to conventional treatment (targeting BP was less than SBP 130 and DBP 80 mmHg) (HR 0.42, p = 0.002). These findings indicate that not only blood glucose control but also other cardiovascular risk factors such as hypertension are important to prevent cerebrovascular events in Japanese patients with T2DM, and that an intensive treatment for hypertension should be more essential in Japanese because cerebrovascular disease is known to be closely associated with blood pressure [16,17].

There are few studies to examine effect of mild hypertension (prehypertension) on atherosclerosis in drug-naïve Japanese diabetic patients. In this study, we thus aimed to examine the association of SBP and DBP with intima-media thickness (IMT), a parameter of atherosclerosis, as well as the impact of prehypertension on the atherosclerosis in drug-naïve Japanese patients with T2DM.

## Subjects and methods

### Subjects

This study was approved by the institutional review board of Shimane University Faculty of Medicine, and the requirement for informed patient consent was waived because no intervention and further examinations were performed. The patients who visited Shimane University hospital for education and treatment of T2DM from 2004–2013 were screened. After excluding subjects who had hepatic or renal dysfunction and any history of CVD and stroke, we included 179 patients with T2DM in this analysis who had never taken any drugs for T2DM, hypertension, dyslipidemia, or atherosclerosis, and underwent the examination by high-resolution B-mode ultrasonography to evaluate IMT of common carotid artery.

### Anthropometric measurements

Body height (cm) was measured using a Martin metal anthropometer to the nearest 0.1 cm according to the standard technique, and body weight (kg) was measured with a medical electronic scale and recorded with 0.05 kg precision with the subject wearing light clothes. Body mass index (BMI) was calculated by the following formula; weight/height in meter^2^. After a more than 5 minutes rest, the systolic blood pressure (SBP) and diastolic blood pressure (DBP) (mmHg) were measured in a supine position using mercury sphygmomanometer for once. A regular-sized cuff appropriate for the Japanese population (arm length 17–32 cm) was used as recommended.

### Measurements of biochemical parameters

After overnight fasting, serum samples were collected. As previously described [18,19], biochemical parameters such as fasting blood glucose (FPG), fasting C-peptide, total cholesterol, triglyceride (TG), high density lipoprotein-cholesterol (HDL-C), and low density lipoprotein-cholesterol (LDL-C) were measured by standard methods. Hemoglobin A1c (HbA1c) was measured by high-performance liquid chromatography. HbA1c values were estimated as NGSP (National Glycohemoglobin Standardization Program) equivalent values. Estimated glomerular filtration rate (eGFR) was calculated using the equation modified coefficients for Japanese [20].

### Carotid IMT measurement

The carotid IMT was measured by B-mode ultrasonographic imaging of the carotid artery was performed using HDI 5000 (Philips, Tokyo, Japan), a high-resolution, real-time ultrasonograph with a 7.5-MHz transducer as previously described [18,19]. Briefly, it was performed in four segments in the bilateral carotid arteries; the 1.5 cm-segment of the internal carotid artery distal to the bifurcation, the bifurcation, the segments of the common carotid artery that were 0 to 1.5 cm, and 1.5 to 3.0 cm proximal to the bifurcation. The following measures were chosen to quantify carotid artery wall thickness: IMT-max; the maximum of 8 sites IMT; IMT-mean mean of the maximum wall thickness of 8 sites (far wall of the bilateral side); and plaque score; sum of the maximum wall thickness of 8sites. Two experienced sonographers independently performed all scans, and averages of the two measures were used in the analysis. A CV of the measurements was 3.55%.

### Statistical analysis

Data were expressed as means ± standard deviation (SD). Pearson’s correlation coefficient was used in univariate analyses. Multiple regression and logistic regression analyses were used for multivariate analysis to adjust confounding factors. Statistical evaluations for differences among three groups were carried out using one-way analysis of variance (ANOVA) followed by the Tukey-Kramer post-hoc test. Receiver operating characteristic (ROC) curves were generated for SBP and DBP as the predictor of IMT. The cut-off point was determined by the area under the ROC curve. Statistical analyses were performed using a statistical computer program StatView (Abacus Concepts, Berkeley, CA) and IBM SPSS version 19 (SPSS Japan Inc., Tokyo, Japan). P < 0.05 was considered to be significant.

## Results

### Background characteristics of subjects and correlation between IMT and various variables

Background characteristics of patients such as demographic, biochemical parameters and IMT are shown in Table 1. First, simple correlation analyses were performed to examine the relationship between IMT and the background characteristics including SBP and DBP (Table 2). IMT-max was significantly and positively correlated with age (r = 0.47, p<0.001) and SBP (r = 0.22, p = 0.004), and negatively with BMI (r = -0.20, p = 0.006), total cholesterol (r = -0.18, p = 0.016), HDL-C (r = -0.16, p = 0.039), and eGFR (r = -0.19, p = 0.009). IMT-mean was significantly and positively correlated with age (r = 0.53, p<0.001), and negatively with total cholesterol (r = -0.29, p = 0.008). Plaque score was significantly and positively correlated with age (r = 0.54, p<0.001) and SBP (r = 0.23, p = 0.010), and negatively with BMI (r = -0.19, p = 0.031).

**Table 1:** Background characteristics of subjects.

Number of subjects (M/F)	179 (121/58)
Age (years)	56.0 ±6.0(y
Duration of diabetes (years)	5.3 ± 6.5
BMI (kg/m^2^)	24.0 ± 5.2
SBP (mmHg)	128 ± 18
DBP (mmHg)	78 ± 12
FPG (mg/dL)	171 ± 69
HbA1c (%)	9.3 ± 3.2
Fasting C-peptide (ng/mL)	1.8 ± 0.8
Total cholesterol (mg/dL)	210 ± 57
Triglyceride (mg/dL)	163 ± 215
HDL-C (mg/dL)	53 ± 16
LDL-C (mg/dL)	126 ±42
eGFR (mL/min/1.73m^2^)	93.3 ± 23.7
IMT-max (mm)	1.8 ± 1.0
IMT-mean (mm)	1.2 ± 0.4
Plaque score	4.7 ± 4.6
Former and current smoking n (%)	97 (54.2)

BMI, body mass index; SBP, systolic blood pressure; DBP, diastolic blood pressure; FPG, fasting blood glucose; HbA1c, hemoglobin A1c; HDL-C, high-density lipoprotein cholesterol; LD-CL, low-density lipoprotein cholesterol; eGFR, estimated glomerular filtration rate; IMT, intima-media thickness

**Table 2:** Correlation between IMT and various variables.

	IMT-max		IMT-mean		Plaque score	
	r	p	r	p	r	p
Age	0.47	<0.001	0.53	<0.001	0.54	<0.001
Duration of diabetes	0.14	0.083	0.14	0.273	0.16	0.101
BMI	-0.20	0.006	-0.17	0.130	-0.19	0.031
SBP	0.22	0.004	0.10	0.365	0.23	0.010
DBP	-0.01	0.925	-0.18	0.100	-0.03	0.722
FPG	0.07	0.352	0.15	0.183	0.14	0.107
HbA1c	0.05	0.496	0.16	0.148	0.13	0.151
Fasting C-peptide	0.01	0.853	0.05	0.678	0.02	0.857
Total cholesterol	-0.18	0.016	-0.29	0.008	-0.17	0.057
Triglyceride	-0.09	0.233	-0.16	0.157	-0.13	0.146
HDL-C	-0.16	0.039	-0.17	0.124	-0.05	0.585
LDL-C	-0.11	0.152	-0.20	0.080	-0.06	0.503
eGFR	-0.19	0.009	-0.08	0.534	-0.17	0.055

### Association of SBP and DBP with IMT

Then, multiple regression analyses adjusted for age, duration of diabetes, BMI, HbA1c, fasting C-peptide, triglyceride, HDL-C, LDL-C, and eGFR were performed (Table 3). SBP was significantly and positively associated with IMT-max (β = 0.28, p<0.001), IMT-mean (β = 0.26, p = 0.047), and plaque score (β = 0.25, p = 0.006).

**Table 3:** Association between blood pressure and IMT.

	IMT-max		IMT-mean		Plaque score	
	β	p	β	p	Β	p
SBP	0.28	<0.001	0.26	0.047	0.25	0.006
DBP	0.11	0.153	-0.05	0.746	0.11	0.236

Adjusted for age, duration of diabetes, BMI, HbA1c, fasting C-peptide, triglyceride, HDL-C, LDL-C, and eGFR

**Fig 1:**
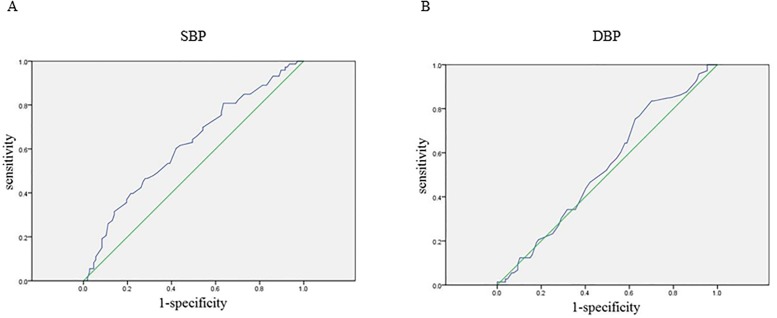
ROC curves to evaluate the cut-off values of SBP and DBP to predict IMT-max above 1.8 mm. (A) SBP, (B) DBP.

Next, we performed ROC analyses to evaluate the cut-off values of SBP and DBP to predict the average of IMT-max of the participants (1.8 mm) (Table 4 and Fig 1). The cut-off values and area under the curve of SBP and DBP were 133.5 mmHg and 0.62 (p = 0.008) and 69.5 mmHg and 0.53 (p = 0.433), respectively. These findings suggest that SBP contributed to atherosclerosis rather than DBP.

**Table 4:** ROC analysis between blood pressure and IMT.

	Cut-off value	sensitivity	specificity	AUC	95% CI	p-value
SBP	133.5	0.47	0.72	0.62	0.53–0.70	0.008
DBP	69.5	0.84	0.30	0.53	0.45–0.62	0.433

AUC, area under the curve; 95% CI, 95% confidence interval

### Association of normotension, prehypertension, and hypertension with IMT

The subjects were divided into three categories according to SBP (normotension; SBP <119 mmHg, prehypertension; SBP 120–139 mmHg, and hypertension >140 mmHg). We compared background characteristics including IMT among subjects with normotension, prehypertension, and hypertension (Table 5). In subjects with prehypertension, BMI, SBP, and DBP were significantly higher (p<0.05, p<0.001, and p<0.001, respectively) and LDL-C was significantly lower (p<0.05) than those in subjects with normotension. In subjects with hypertension, BMI, SBP, DBP, fasting C-peptide, and IMT-max were significantly higher (p<0.01, p<0.001, p<0.001, p<0.05, and p<0.01, respectively) and LDL-C was significantly lower (p<0.05) than those in subjects with normotension. In subjects with hypertension, SBP, DBP, HDL-C, IMT-max, and plaque score were significantly higher (p<0.01, p<0.001, p<0.05, p<0.01, and p<0.05, respectively) than those in subjects with prehypertension.

**Table 5:** Comparison of various variables among subjects with normotension, prehypertension, and hypertension.

SBP	Normotension <119 mmHg	Prehypertension 120–139 mmHg		Hypertension >140 mmHg	
Number of subjects (M/F)	53 (29/24)	79 (60/19)		47 (32/15)	
Age (years)	58.1 ± 11.2	53.9 ± 13.6		57.3 ± 12.6	
Duration of diabetes (years)	5.1 ± 5.7	5.5 ± 7.1		5.2 ± 6.3	
BMI (kg/m^2^)	22.2 ± 3.5	24.6 ± 4.7	*	25.1 ± 6.9	**
SBP (mmHg)	108 ± 8	128 ± 6	***	150 ± 11	***
					×××
DBP (mmHg)	71 ± 9	78 ± 8	***	85 ± 15	***
					×××
FPG (mg/dL)	168 ± 71	167 ± 68		181 ± 66	
HbA1c (%)	9.1 ± 3.1	9.2 ± 3.4		9.5 ± 2.9	
Fasting C-peptide (ng/mL)	1.6 ± 0.6	1.8 ± 0.8		2.0 ± 1.0	*
Total cholesterol (mg/dL)	217 ± 54	202 ± 50		215 ± 68	
Triglyceride (mg/dL)	150 ± 90	143 ± 93		212 ± 89	
HDL-C (mg/dL)	52 ± 15	51 ± 15		58 ± 19	×
LDL-C (mg/dL)	137 ± 46	122 ± 40	*	120 ± 41	*
eGFR (mL/min/1,73m^2^)	93.5 ± 22.4	93.1 ± 21.5		93.3 ± 28.6	
IMT-max (mm)	1.6 ± 0.8	1.7 ± 0.9		2.2 ± 1.3	**
					××
IMT-mean (mm)	1.2 ± 0.4	1.2 ± 0.4		1.2 ± 0.4	
Plaque score	4.3 ± 4.6	4.1 ± 4.2		6.1 ± 5.0	×
Former and current smoking, n (%)	23 (43.4)	50 (63.3)		24 (51.1)	

*; p<0.05,

**; p<0.01,

***; p<0.001 v.s normotension

^×^; p<0.05,

^××^; p<0.01,

^×××^; p<0.001 v.s prehypertension

Because several background characteristics were different among the hypertensive status, we performed multiple logistic regression analyses adjusted for age, gender, duration of diabetes, BMI, HbA1c, fasting C-peptide, triglyceride, HDL-C, LDL-C, eGFR, and smoking to examine the independent association between hypertensive status and IMT-max (Table 6). Prehypertension and hypertension were significantly and positively associated with IMT-max above 1.8 mm [(odds ratio (OR) = 0.345, 95% confidence interval (CI) 1.11–10.76, p = 0.033 and OR = 7.29, 95%CI 1.99–26.78, p = 0.003].

**Table 6:** Association between hypertension status and IMT.

	IMT-max (above 1.8 mm)		
	Odds ratio	95% CI	p-value
Normotension	1.00	-	-
Prehypertension	3.45	1.11–10.76	0.033
Hypertension	7.29	1.99–26.78	0.003

Adjusted for age, duration of diabetes, BMI, HbA1c, fasting C-peptide, triglyceride, HDL-C, LDL-C, eGFR, and smoking

## Discussion

The present study showed that higher SBP was significantly and linearly associated with increased IMT independently of conventional risk factors of atherosclerosis in drug-naïve patients with T2DM, whereas DBP was not associated with IMT. ROC curve showed that the cut-off value of SBP to detect over average IMT of the participants was 133.5 mmHg. Moreover, prehypertension categorized by SBP was also associated with IMT. These findings suggest that SBP is an independent predictor of increased IMT; thus, we have to pay careful attention to atherosclerotic diseases even when drug-naïve diabetic patients have SBP 120–139 mmHg.

In this study, we used carotid IMT as a surrogate marker of atherosclerosis. IMT is commonly used as a noninvasive examination for the assessment of degree of atherosclerosis. Numerous studies have shown that increased IMT is a strong predictor of CVD and ischemic stroke [20–23]. In this study, none of the subjects had a history of CVD and stroke because we focused on the association between BP and IMT in drug-naïve patients with T2DM. Furthermore, because this is a cross-sectional study, we can’t conclude whether prehypertension is associated with cardio- and cerebrovascular events. We are thus conducting a prospective study to examine whether prehypertension is a therapeutic target in Japanese diabetic patients. However, a recent multifactorial intervention study has shown that intensive treatment including anti-hypertension therapy significantly reduced the risk of cerebrovascular events in Japanese patients with T2DM [15], although diabetes and dyslipidemia were also treated. Taken together, these findings suggest that the intensive therapy targeting SBP <120 mmHg is important for Japanese patients to prevent ischemic stroke.

Because there are ethnic differences in the frequency of CVD and stroke as well as in the impact of BP on these diseases between Asian and Western people [13,14], it is an important task to establish clinical evidence in Asian patients. Several epidemiological studies have shown that prehypertension is a risk factor of CVD in Asian [9–11]. The findings of this study using Japanese patients are consistent with the previous studies. However, there are few interventional studies to examine the effects of anti-hypertensive treatments for “prehypertension” on parameters of atherosclerosis as well as CVD and stroke events in Japanese patients with T2DM. Therefore, we have to approach this issue to clarify whether or not the treatment of prehypertension is beneficial for patients with T2DM in future.

This study has some limitations. First, we examined only patients who visited our hospital, which is a tertiary center for treatment of diabetes mellitus. Therefore, the subjects included in this analysis might have relatively severe states of T2DM and might not be representative of Japanese patients. Indeed, the mean of IMT-max was 1.8 mm of the participants, which might be quite greater compared to healthy subjects. Although we used IMT-max above 1.8 mm as a cut-off value of the presence of atherosclerosis in this study, there might be more appropriate cut-off point in other population. Second, the conclusion of this study is weakened because of its cross-sectional design. It is therefore necessary to conduct longitudinal studies to confirm the present findings.

In conclusion, the present study showed that SBP was independently and linearly associated with increased IMT in drug-naïve Japanese patients with T2DM, suggesting that the lower SBP is the better for prevention of atherosclerotic diseases, and that we should take the presence of atherosclerosis into account in case of diabetic patients with prehypertension. However, further studies are necessary to define the management strategy for prehypertension in patients with T2DM.
